# Benzene-1,3-dicarb­oxy­lic acid–1,2-bis­(4-pyrid­yl)ethene (1/1)

**DOI:** 10.1107/S1600536811043418

**Published:** 2011-10-29

**Authors:** Dong Liu, Ni-Ya Li

**Affiliations:** a College of Chemistry and Materials Science, Huaibei Normal University, Huaibei 235000, Anhui, People’s Republic of China

## Abstract

In the title compound, C_12_H_10_N_2_·C_8_H_6_O_4_, the asymmetric unit contains two halves of 1,2-bis­(4-pyrid­yl)ethene (bpe) mol­ecules and one benzene-1,3-dicarb­oxy­lic acid (1,3-H_2_BDC) mol­ecule. These bpe and 1,3-H_2_BDC mol­ecules are linked by classical O—H⋯N hydrogen bonds, forming an extended one-dimensional zigzag chain. Each chain is further linked with neighboring ones by π–π inter­actions between the pyridine and aromatic rings [centroid–centroid distances = 3.9306 (15) Å] and the pyridine rings of pairs of symmetry-related mol­ecules [centroid–centroid distances = 3.5751 (15), 3.7350 (15) and 3.6882 (15) Å], with the formation of a three-dimensional supra­molecular framework.

## Related literature

For structures and properties of self-assembled supramolecular compounds, see: Lehn (1990 ▶). For hydrogen-bonding inter­actions and π–π inter­actions in supramolecular compounds, see: Biradha (2003 ▶); Shan & Jones (2003 ▶); Weyna *et al.* (2009 ▶).
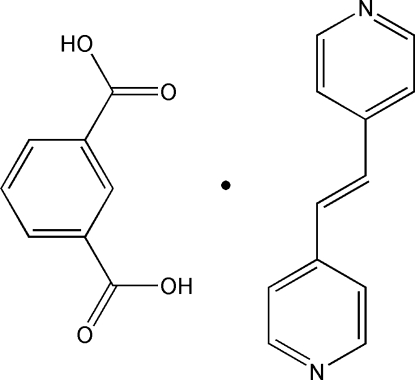


## Experimental

### 

#### Crystal data

C_12_H_10_N_2_·C_8_H_6_O_4_
*M* *_r_* = 348.35
Triclinic, 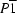

*a* = 6.8331 (14) Å
*b* = 6.8804 (14) Å
*c* = 18.618 (4) Å
α = 99.47 (3)°
β = 93.87 (3)°
γ = 102.69 (3)°
*V* = 837.4 (3) Å^3^
*Z* = 2
Mo *K*α radiation
μ = 0.10 mm^−1^
*T* = 223 K
0.40 × 0.40 × 0.35 mm

#### Data collection

Rigaku Mercury CCD diffractometer
Absorption correction: multi-scan (*REQAB*; Jacobson, 1998 ▶) *T*_min_ = 0.962, *T*_max_ = 0.967
8280 measured reflections
3054 independent reflections
2153 reflections with *I* > 2σ(*I*)
*R*_int_ = 0.037

#### Refinement

*R*[*F* ^2^ > 2σ(*F* ^2^)] = 0.051
*wR*(*F* ^2^) = 0.137
*S* = 1.06
3054 reflections
238 parameters
H-atom parameters constrained
Δρ_max_ = 0.23 e Å^−3^
Δρ_min_ = −0.19 e Å^−3^

### 

Data collection: *CrystalClear* (Rigaku, 2001 ▶); cell refinement: *CrystalClear*; data reduction: *CrystalStructure* (Rigaku/MSC, 2004 ▶); program(s) used to solve structure: *SHELXTL* (Sheldrick, 2008 ▶); program(s) used to refine structure: *SHELXTL*; molecular graphics: *SHELXTL*; software used to prepare material for publication: *SHELXTL* and *PLATON* (Spek, 2009 ▶).

## Supplementary Material

Crystal structure: contains datablock(s) I, global. DOI: 10.1107/S1600536811043418/rk2306sup1.cif

Structure factors: contains datablock(s) I. DOI: 10.1107/S1600536811043418/rk2306Isup2.hkl

Supplementary material file. DOI: 10.1107/S1600536811043418/rk2306Isup3.cml

Additional supplementary materials:  crystallographic information; 3D view; checkCIF report

## Figures and Tables

**Table 1:** Hydrogen-bond geometry (Å, °)

*D*—H⋯*A*	*D*—H	H⋯*A*	*D*⋯*A*	*D*—H⋯*A*
O1—H1⋯N1^i^	0.83	1.77	2.597 (2)	176
O3—H3⋯N2	0.83	1.79	2.618 (2)	176

Symmetry code: (i) 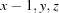.

**Fig. 1.**
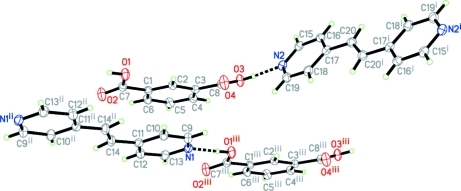
The structure of the title compound, showing the atom-numbering scheme. Displacement ellipsoids are presented at the 30% probability level. Symmetry codes: (i) -x+2, -y+2, -z+1; (ii): -x, -y+1, -z+2; (iii) x+1, y, z.

**Fig. 2.**
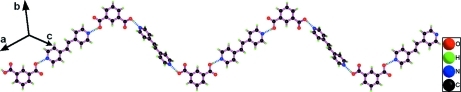
The one-dimensional zigzag chain linked by hydrogen-bonding interactions. The blue dashed lines represent the hydrogen-bonds.

**Fig. 3.**
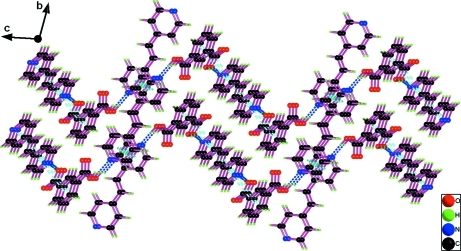
The three-dimensional supramolecular framework linked by hydrogen-bonding interactions and π–π interactions. The blue and cyan dashed lines represent the hydrogen-bonds and π–π interactions, respectively.

## supplementary crystallographic information

### Comment

In the past decades, the supramolecular synthesis of multicomponent organic materials has attracted considerable attention due to their functional properties (Lehn, 1990). The facile way of synthesizing these co-crystals is to employ the components containing complementary functional groups such as pyridine and carboxylic acid. Owing to the hydrogen-bonds and π–π stacking between these types of groups, several multicomponent cocrystals containing various network geometries were prepared using these two functional groups (Biradha, 2003; Shan & Jones, 2003; Weyna *et al.*, 2009).

The hydrothermal reaction of 1,2-bis(4-pyridyl)ethene (bpe) with benzene-1,3-dicarboxylic acid (1,3-H_2_BDC) resulted in the cocrystals of C_12_H_10_N_2_.C_8_H_6_O_4_, I. In I, the asymmetric unit is formed by two halves of bpe molecules and one 1,3-H_2_BDC molecule (Fig. 1). These molecular units are linked by classical O–H···N hydrogen-bonds (O1–H1···N1^iii^, O1···N1^iii^ = 2.597 (2)Å; O3–H3···N2, O3···N2 = 2.618 (2)Å) forming an extended one-dimensional zigzag chain (Table 1, Fig. 2). Furthermore, the adjacent one-dimensional chains are interconnected each other through π–π interactions between pairs of molecules [*Cg*1···*Cg*1^iv^ = 3.5751 (15)Å; *Cg*1···*Cg*3 = 3.9306 (15)Å; *Cg*2···*Cg*2^v^ = 3.7350 (15)Å; *Cg*2···*Cg*2^vi^ = 3.7350 (15)Å form a three-dimensional framework (Fig. 3). The *Cg*1, *Cg*2 and *Cg*3, are the centroids of the rings N1/C9-C13, N2/C15-C19 and C1-C6. Symmetry codes: (iii) -*x*+1, *y*, *z*; (iv) -*x*+1, -*y*+1, -*z*+2; (v) -*x*+1, -*y*+1, -*z*+1; (vi) -*x*+2, -*y*+1, -*z*+1 .

### Experimental

To a 10 mL Pyrex glass tube was loaded 1,2-bis(4-pyridyl)ethene (18 mg, 0.1 mmol), benzene-1,3-dicarboxylic acid (17 mg, 0.1 mmol) and 3 ml of H_2_O. The tube was sealed and heated in an oven to 423 K for three days, and then cooled to ambient temperature at the rate of 5 K h^-1^ to form yellow crystals.

### Refinement

All H atoms were placed in geometrically idealized positions (C–H = 0.94Å for phenyl, pyridyl and vinyl groups, O–H = 0.83Å for OH group) and constrained to ride on their parent atoms with *U*_iso_(H) = 1.2*U*_eq_(C) and *U*_iso_(H) = 1.5*U*_eq_(O).

### Crystal data

**Table:**

C_12_H_10_N_2_·C_8_H_6_O_4_	*Z* = 2
*M**_r_* = 348.35	*F*(000) = 364
Triclinic, *P*1	*D*_x_ = 1.382 Mg m^−^^3^
Hall symbol: -P 1	Mo *K*α radiation, λ = 0.71073 Å
*a* = 6.8331 (14) Å	Cell parameters from 2779 reflections
*b* = 6.8804 (14) Å	θ = 3.1–25.4°
*c* = 18.618 (4) Å	µ = 0.10 mm^−^^1^
α = 99.47 (3)°	*T* = 223 K
β = 93.87 (3)°	Block, yellow
γ = 102.69 (3)°	0.40 × 0.40 × 0.35 mm
*V* = 837.4 (3) Å^3^	

### Data collection

**Table:**

Rigaku Mercury CCD diffractometer	3054 independent reflections
Radiation source: fine-focus sealed tube	2153 reflections with *I* > 2σ(*I*)
graphite	*R*_int_ = 0.037
φ and ω scans	θ_max_ = 25.3°, θ_min_ = 3.1°
Absorption correction: multi-scan (*REQAB*; Jacobson, 1998)	*h* = −8→8
*T*_min_ = 0.962, *T*_max_ = 0.967	*k* = −7→8
8280 measured reflections	*l* = −22→21

### Refinement

**Table:**

Refinement on *F*^2^	Secondary atom site location: difference Fourier map
Least-squares matrix: full	Hydrogen site location: inferred from neighbouring sites
*R*[*F*^2^ > 2σ(*F*^2^)] = 0.051	H-atom parameters constrained
*wR*(*F*^2^) = 0.137	*w* = 1/[σ^2^(*F*_o_^2^) + (0.0616*P*)^2^ + 0.1982*P*] where *P* = (*F*_o_^2^ + 2*F*_c_^2^)/3
*S* = 1.06	(Δ/σ)_max_ < 0.001
3054 reflections	Δρ_max_ = 0.23 e Å^−^^3^
238 parameters	Δρ_min_ = −0.19 e Å^−^^3^
0 restraints	Extinction correction: *SHELXTL* (Sheldrick, 2008), Fc^*^=kFc[1+0.001xFc^2^λ^3^/sin(2θ)]^-1/4^
Primary atom site location: structure-invariant direct methods	Extinction coefficient: 0.028 (4)

### Special details

**Table:**

Geometry. All s.u.'s (except the s.u. in the dihedral angle between two l.s. planes) are estimated using the full covariance matrix. The cell s.u.'s are taken into account individually in the estimation of s.u.'s in distances, angles and torsion angles; correlations between s.u.'s in cell parameters are only used when they are defined by crystal symmetry. An approximate (isotropic) treatment of cell s.u.'s is used for estimating s.u.'s involving l.s. planes.
Refinement. Refinement of *F*^2^ against ALL reflections. The weighted *R*-factor *wR* and goodness of fit *S* are based on *F*^2^, conventional *R*-factors *R* are based on *F*, with *F* set to zero for negative *F*^2^. The threshold expression of *F*^2^ > σ(*F*^2^) is used only for calculating *R*-factors(gt) *etc*. and is not relevant to the choice of reflections for refinement. *R*-factors based on *F*^2^ are statistically about twice as large as those based on *F*, and *R*-factors based on ALL data will be even larger.

### Fractional atomic coordinates and isotropic or equivalent isotropic displacement parameters (Å^2^)

**Table:**

	*x*	*y*	*z*	*U*_iso_*/*U*_eq_	
O1	−0.1898 (3)	0.1743 (2)	0.81468 (9)	0.0437 (5)	
H1	−0.2835	0.1999	0.8378	0.065*	
O2	−0.2446 (3)	−0.0994 (3)	0.86694 (10)	0.0528 (5)	
O3	0.5207 (2)	0.0878 (2)	0.62885 (9)	0.0417 (4)	
H3	0.5796	0.1779	0.6079	0.063*	
O4	0.4236 (3)	0.3460 (2)	0.69323 (9)	0.0495 (5)	
N1	0.5203 (3)	0.2721 (3)	0.88516 (10)	0.0348 (5)	
N2	0.6910 (3)	0.3812 (3)	0.56392 (10)	0.0353 (5)	
C1	0.0121 (3)	−0.0583 (3)	0.78717 (11)	0.0306 (5)	
C2	0.1342 (3)	0.0735 (3)	0.75112 (11)	0.0292 (5)	
H2	0.1109	0.2030	0.7507	0.035*	
C3	0.2909 (3)	0.0166 (3)	0.71557 (11)	0.0283 (5)	
C4	0.3240 (3)	−0.1741 (3)	0.71652 (11)	0.0346 (5)	
H4	0.4293	−0.2141	0.6925	0.041*	
C5	0.2028 (4)	−0.3062 (3)	0.75262 (12)	0.0403 (6)	
H5	0.2260	−0.4357	0.7530	0.048*	
C6	0.0478 (4)	−0.2489 (3)	0.78806 (12)	0.0369 (6)	
H6	−0.0337	−0.3389	0.8128	0.044*	
C7	−0.1539 (3)	0.0031 (3)	0.82700 (12)	0.0347 (5)	
C8	0.4193 (3)	0.1663 (3)	0.67866 (11)	0.0327 (5)	
C9	0.4644 (3)	0.4451 (4)	0.88158 (12)	0.0380 (6)	
H9	0.5339	0.5335	0.8533	0.046*	
C10	0.3099 (3)	0.4988 (3)	0.91739 (12)	0.0372 (6)	
H10	0.2752	0.6216	0.9132	0.045*	
C11	0.2046 (3)	0.3725 (3)	0.95979 (11)	0.0334 (5)	
C12	0.2643 (3)	0.1941 (4)	0.96377 (12)	0.0390 (6)	
H12	0.1989	0.1041	0.9923	0.047*	
C13	0.4199 (3)	0.1495 (4)	0.92569 (12)	0.0379 (6)	
H13	0.4568	0.0270	0.9284	0.046*	
C14	0.0399 (3)	0.4214 (4)	1.00081 (13)	0.0391 (6)	
H14	−0.0139	0.3314	1.0313	0.047*	
C15	0.7001 (3)	0.3777 (3)	0.49225 (12)	0.0342 (5)	
H15	0.6506	0.2538	0.4597	0.041*	
C16	0.7789 (3)	0.5474 (3)	0.46388 (12)	0.0332 (5)	
H16	0.7805	0.5380	0.4130	0.040*	
C17	0.8558 (3)	0.7326 (3)	0.51039 (12)	0.0324 (5)	
C18	0.8411 (3)	0.7367 (4)	0.58473 (12)	0.0383 (6)	
H18	0.8865	0.8590	0.6184	0.046*	
C19	0.7600 (3)	0.5615 (4)	0.60859 (12)	0.0404 (6)	
H19	0.7522	0.5677	0.6591	0.049*	
C20	0.9500 (3)	0.9104 (3)	0.48071 (12)	0.0353 (5)	
H20	0.9390	0.8969	0.4294	0.042*	

### Atomic displacement parameters (Å^2^)

**Table:**

	*U*^11^	*U*^22^	*U*^33^	*U*^12^	*U*^13^	*U*^23^
O1	0.0436 (11)	0.0443 (10)	0.0532 (10)	0.0215 (8)	0.0243 (8)	0.0151 (8)
O2	0.0570 (12)	0.0512 (11)	0.0617 (11)	0.0181 (9)	0.0331 (9)	0.0247 (9)
O3	0.0466 (11)	0.0339 (9)	0.0481 (10)	0.0108 (8)	0.0249 (8)	0.0073 (8)
O4	0.0646 (12)	0.0296 (10)	0.0625 (11)	0.0167 (8)	0.0341 (9)	0.0129 (8)
N1	0.0284 (11)	0.0395 (11)	0.0360 (10)	0.0086 (9)	0.0060 (8)	0.0037 (9)
N2	0.0294 (11)	0.0360 (11)	0.0406 (11)	0.0080 (8)	0.0077 (8)	0.0058 (9)
C1	0.0326 (12)	0.0319 (12)	0.0271 (11)	0.0088 (10)	0.0034 (9)	0.0033 (9)
C2	0.0339 (13)	0.0262 (11)	0.0285 (11)	0.0099 (10)	0.0037 (9)	0.0038 (9)
C3	0.0305 (12)	0.0267 (11)	0.0278 (11)	0.0087 (9)	0.0033 (9)	0.0024 (9)
C4	0.0389 (14)	0.0348 (13)	0.0330 (12)	0.0150 (11)	0.0077 (10)	0.0049 (10)
C5	0.0506 (16)	0.0309 (13)	0.0443 (14)	0.0174 (12)	0.0113 (11)	0.0074 (11)
C6	0.0436 (15)	0.0317 (12)	0.0378 (13)	0.0094 (11)	0.0097 (10)	0.0103 (10)
C7	0.0343 (13)	0.0349 (13)	0.0348 (12)	0.0085 (11)	0.0055 (10)	0.0049 (10)
C8	0.0341 (13)	0.0335 (13)	0.0324 (12)	0.0127 (10)	0.0082 (9)	0.0035 (10)
C9	0.0341 (13)	0.0397 (14)	0.0416 (13)	0.0083 (11)	0.0118 (10)	0.0091 (11)
C10	0.0361 (14)	0.0354 (13)	0.0423 (13)	0.0121 (11)	0.0096 (10)	0.0060 (11)
C11	0.0289 (12)	0.0397 (13)	0.0293 (11)	0.0080 (10)	0.0022 (9)	0.0003 (10)
C12	0.0383 (14)	0.0419 (14)	0.0396 (13)	0.0112 (11)	0.0115 (10)	0.0103 (11)
C13	0.0353 (14)	0.0399 (13)	0.0413 (13)	0.0149 (11)	0.0059 (10)	0.0063 (11)
C14	0.0341 (13)	0.0451 (14)	0.0398 (12)	0.0111 (11)	0.0133 (10)	0.0067 (11)
C15	0.0281 (12)	0.0332 (13)	0.0408 (13)	0.0094 (10)	0.0055 (9)	0.0018 (10)
C16	0.0299 (12)	0.0346 (13)	0.0356 (12)	0.0105 (10)	0.0035 (9)	0.0037 (10)
C17	0.0246 (12)	0.0319 (12)	0.0394 (12)	0.0067 (10)	0.0016 (9)	0.0037 (10)
C18	0.0349 (13)	0.0347 (13)	0.0385 (13)	0.0006 (11)	0.0038 (10)	−0.0021 (11)
C19	0.0360 (14)	0.0473 (15)	0.0344 (12)	0.0041 (11)	0.0074 (10)	0.0036 (11)
C20	0.0332 (13)	0.0345 (12)	0.0387 (13)	0.0082 (10)	0.0028 (10)	0.0083 (10)

### Geometric parameters (Å, °)

**Table:**

O1—C7	1.307 (3)	C9—C10	1.371 (3)
O1—H1	0.8300	C9—H9	0.9400
O2—C7	1.215 (3)	C10—C11	1.387 (3)
O3—C8	1.310 (2)	C10—H10	0.9400
O3—H3	0.8300	C11—C12	1.387 (3)
O4—C8	1.215 (3)	C11—C14	1.470 (3)
N1—C13	1.333 (3)	C12—C13	1.378 (3)
N1—C9	1.338 (3)	C12—H12	0.9400
N2—C15	1.336 (3)	C13—H13	0.9400
N2—C19	1.343 (3)	C14—C14^i^	1.318 (5)
C1—C2	1.383 (3)	C14—H14	0.9400
C1—C6	1.387 (3)	C15—C16	1.378 (3)
C1—C7	1.495 (3)	C15—H15	0.9400
C2—C3	1.390 (3)	C16—C17	1.390 (3)
C2—H2	0.9400	C16—H16	0.9400
C3—C4	1.383 (3)	C17—C18	1.390 (3)
C3—C8	1.490 (3)	C17—C20	1.463 (3)
C4—C5	1.382 (3)	C18—C19	1.369 (3)
C4—H4	0.9400	C18—H18	0.9400
C5—C6	1.381 (3)	C19—H19	0.9400
C5—H5	0.9400	C20—C20^ii^	1.333 (4)
C6—H6	0.9400	C20—H20	0.9400
			
C7—O1—H1	109.5	C9—C10—H10	119.9
C8—O3—H3	109.5	C11—C10—H10	119.9
C13—N1—C9	117.51 (19)	C10—C11—C12	116.8 (2)
C15—N2—C19	116.9 (2)	C10—C11—C14	123.4 (2)
C2—C1—C6	119.4 (2)	C12—C11—C14	119.8 (2)
C2—C1—C7	121.13 (19)	C13—C12—C11	119.7 (2)
C6—C1—C7	119.4 (2)	C13—C12—H12	120.2
C1—C2—C3	120.74 (19)	C11—C12—H12	120.2
C1—C2—H2	119.6	N1—C13—C12	123.1 (2)
C3—C2—H2	119.6	N1—C13—H13	118.5
C4—C3—C2	119.2 (2)	C12—C13—H13	118.5
C4—C3—C8	122.45 (19)	C14^i^—C14—C11	126.6 (3)
C2—C3—C8	118.30 (18)	C14^i^—C14—H14	116.7
C5—C4—C3	120.3 (2)	C11—C14—H14	116.7
C5—C4—H4	119.9	N2—C15—C16	123.0 (2)
C3—C4—H4	119.9	N2—C15—H15	118.5
C6—C5—C4	120.3 (2)	C16—C15—H15	118.5
C6—C5—H5	119.9	C15—C16—C17	120.1 (2)
C4—C5—H5	119.9	C15—C16—H16	120.0
C5—C6—C1	120.1 (2)	C17—C16—H16	120.0
C5—C6—H6	120.0	C16—C17—C18	116.7 (2)
C1—C6—H6	120.0	C16—C17—C20	120.1 (2)
O2—C7—O1	124.3 (2)	C18—C17—C20	123.1 (2)
O2—C7—C1	121.9 (2)	C19—C18—C17	119.6 (2)
O1—C7—C1	113.81 (19)	C19—C18—H18	120.2
O4—C8—O3	123.6 (2)	C17—C18—H18	120.2
O4—C8—C3	121.53 (19)	N2—C19—C18	123.7 (2)
O3—C8—C3	114.84 (18)	N2—C19—H19	118.2
N1—C9—C10	122.7 (2)	C18—C19—H19	118.2
N1—C9—H9	118.6	C20^ii^—C20—C17	126.3 (3)
C10—C9—H9	118.6	C20^ii^—C20—H20	116.8
C9—C10—C11	120.2 (2)	C17—C20—H20	116.8

Symmetry codes: (i) −*x*, −*y*+1, −*z*+2; (ii) −*x*+2, −*y*+2, −*z*+1.

### Hydrogen-bond geometry (Å, °)

**Table:**

*D*—H···*A*	*D*—H	H···*A*	*D*···*A*	*D*—H···*A*
O1—H1···N1^iii^	0.83	1.77	2.597 (2)	176.
O3—H3···N2	0.83	1.79	2.618 (2)	176.

Symmetry codes: (iii) *x*−1, *y*, *z*.
